# Biocontrol Potential of Selected Lamiaceae Species Against *Ralstonia solanacearum* (Smith) Causing Wilting in Hot Pepper in Ethiopia

**DOI:** 10.1155/tswj/9940237

**Published:** 2026-03-04

**Authors:** Yitayih Dessie, Destaw Mullualem, Belsti Atnkut, Alemu Tsega, Bulti Kumera, Misganaw Liyew, Ashebir Awoke

**Affiliations:** ^1^ Department of Biology, College of Natural and Computational Sciences, Injibara University, Injibara, Ethiopia, inu.edu.et; ^2^ Graduate School Chinese Academy of Agricultural Sciences (CAAS), Beijing, China; ^3^ Department of Biology, College of Science, Bahir Dar University, Bahir Dar, Ethiopia, bdu.edu.et; ^4^ Department of Biology, College of Natural and Computational Sciences, Mizan-Tepi University, Mizan, Ethiopia, mtu.edu.et

**Keywords:** inhibition zone, Lamiaceae, MBC, medicinal plants, MIC, *phytochemicals*, *Ralstonia solanacearum*

## Abstract

**Background:**

Hot pepper (*Capsicum annuum* L.) is a significant crop worldwide, including in Ethiopia, but its yield is radically dropping owing to several biotic factors such as bacterial wilting, necessitating the development of environmentally benign plant‐based natural treatments to address these challenges. Therefore, this study seeks to evaluate the biocontrol potential of selected Lamiaceae species against *Ralstonia solanacearum* in hot pepper cultivation.

**Methods:**

Ethanolic leaf extracts from six plant species were prepared using the maceration technique and characterized through qualitative and quantitative phytochemical analyses. *R. solanacearum* was isolated from infected hot peppers and identified using biochemical tests. The antibacterial activity of plant crude extracts was assessed in vitro against *R. solanacearum* using the agar well diffusion assay. The broth microdilution method was used to determine the minimum inhibitory concentrations (MIC) and minimum bactericidal concentrations (MBC). The data were analyzed using analysis of variance (ANOVA).

**Results:**

All of the examined plants′ ethanol extracts contain alkaloids, phenolics, and terpenoids. *Mentha spicata* had the highest total phenolic content (184.5 ± 0.5 mg GAE/g dry sample), whereas *Salvia schimperi* had the highest total flavonoid content (144.3 ± 0.3 mg CE/g). Plant extracts revealed a dose‐dependent gradual increase in the inhibitory zone. *M. spicata* extract had the highest antibacterial activity (18.33 ± 0.57), followed by *S. schimperi* (16 ± 1) at 50 mg/mL. The zone of inhibition differed significantly among the analyzed plant extracts (*p* < 0.05). *M. spicata* had a lower MIC (6.25 mg/mL) and MBC (12.5 mg/mL).

**Conclusion:**

The Lamiaceae family holds significant potential as a biocontrol agent against *R. solanacearum*, as antibacterial compounds were found in the extract. Future research should focus on validating *M. spicata* and *S. schimperi* extracts through in vivo field trials to develop sustainable, plant‐based biopesticides for Ethiopian pepper farmers.

## 1. Introduction

Hot pepper (*Capsicum* spp.) is a widely cultivated crop known for its culinary, nutritional, and economic importance globally [1, 2]. Renowned for its pungent flavor and vibrant color, hot pepper is a staple in various cuisines and plays a crucial role in the diets of millions [1]. Beyond its culinary appeal, hot pepper offers significant health benefits, including anti‐inflammatory, antioxidant, and antimicrobial properties attributed to its active compound, capsaicin [3, 4].

In recent years, global demand for hot peppers has surged, driven by evolving international culinary trends, an increased consumer interest in health‐conscious diets, and the expansion of the global spice trade [3]. This heightened demand has spurred expanded cultivation across Asia, Africa, and the Americas [5, 6]. In many developing nations, hot pepper farming serves as a dual‐purpose endeavor: It ensures food security and provides a critical source of income for smallholder farmers, thereby bolstering local economies [7].

Despite its economic importance, pepper production is increasingly threatened by biotic stressors, most notably pests and diseases [8]. Among these, *Ralstonia solanacearum* is recognized as one of the most destructive plant pathogens [9]. A Gram‐negative bacterium, *R. solanacearum* is the causal agent of bacterial wilt in over 200 plant species, including high‐value crops such as tomatoes, potatoes, and hot peppers [10]. The pathogen′s virulence is attributed to its ability to invade host vascular tissues, where rapid multiplication and the production of extracellular polysaccharides lead to systemic wilting and devastating yield losses [11].

Bacterial wilt, caused by the *Ralstonia solanacearum* species complex (RSSC), is one of the most devastating constraints on global hot pepper (*Capsicum* spp.) production [12]. Ranked as the second most destructive bacterial plant pathogen worldwide, it causes yield losses ranging from 20% to 100% depending on the environment and cultivar [13]. With an estimated annual economic impact exceeding 1 billion globally, the pathogen affects roughly 1.5 million hectares across 80 countries [14]. In major production regions, such as Ethiopia and China, disease incidence frequently reaches 100%, forcing smallholder farmers to abandon infested fields due to the pathogen′s high soil persistence [15].

In Ethiopia, hot pepper farming holds substantial economic and nutritional significance, supporting the livelihoods of many smallholder farmers [16]. The Amhara region is particularly noted for its hot pepper production. Powdered hot pepper is a key ingredient in the traditional sauce known as “wot”, and the average Ethiopian adult consumes approximately 15 g of hot pepper daily, surpassing consumption levels of tomatoes and most other vegetables [17]. It is an essential component of the Ethiopian diet. Peppers are utilized in local dishes in different forms, such as “Qaria” (green pods), “berberie” **(**a fine powder made from dried hot pepper fruits), and “mitmita” which consists of small, very pungent fruits.

Despite its importance, hot pepper production in Ethiopia is severely impacted by biotic stresses, with bacterial wilt caused by *R. solanacearum* being particularly devastating [18]. Current control measures primarily rely on chemical methods, which can pose risks to human health and the environment [19–22]. Therefore, there is an urgent need for sustainable and environmentally friendly alternatives to manage this pathogen, especially in a country where agriculture is a cornerstone of the economy.

This study explores the potential of Lamiaceae plants as biocontrol agents against *R. solanacearum*. Unlike previous research that focused on the chemical properties and traditional uses of these plants, this investigation specifically addresses the threat posed by *R. solanacearum* to hot pepper cultivation. Conducted within Ethiopia′s unique agricultural context, the study examines local Lamiaceae species to identify culturally relevant and sustainable biocontrol methods. It also looks at how these plants stop the pathogen from spreading, such as through allelopathic effects.

The Lamiaceae family, which includes aromatic herbs like basil, mint, and thyme, has gained attention for its potential in biocontrol applications [23, 24]. Many members possess antimicrobial properties due to essential oils and secondary metabolites that may inhibit pathogen growth [25–28]. Preliminary studies have suggested that certain Lamiaceae species could effectively suppress *R. solanacearum*, presenting a promising approach for managing various crop diseases [24, 29–31]. We hypothesize that extracts from the Lamiaceae family will demonstrate significant antibacterial activity against this bacterium, which is a major pathogen in Ethiopian hot pepper crops. By investigating the mechanisms of action, this study is aimed at providing insights into the sustainable use of these plants in agricultural practices, potentially leading to new techniques for managing bacterial wilt and improving crop resilience and farmer livelihoods, particularly in Ethiopia.

## 2. Methods and Materials

### 2.1. Plant Materials Collection

**Figure 1:**
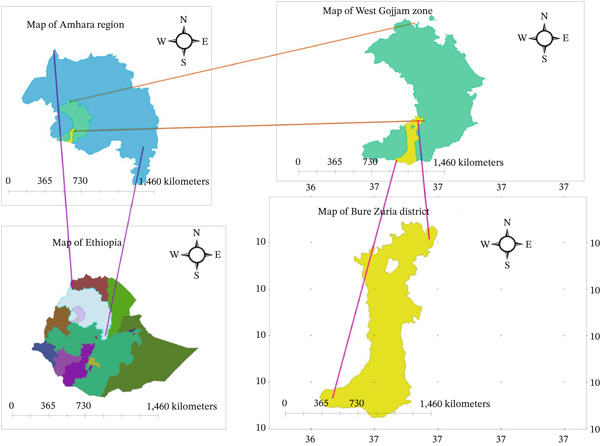
Map of the study area illustrating the primary regions of pepper cultivation and the documented locations of key medicinal plant species identified during the field survey.

Medicinal plants were chosen after conducting a reconnaissance survey based on their traditional use in treating bacterial diseases and antimicrobial activity reports in the literature. Thus, the most reported plants were selected for biocontrol investigation. The leaves of six selected plant species from the Lamiaceae family were collected from Bure Zuria District, around 403 km northwest of Addis Ababa, at points of latitude 10°18 ^′^N–10°49 ^′^29 ^″^N and longitude 36°52 ^′^1 ^″^E–37°7 ^′^9 ^″^E (Figure 1).

**Figure 2:**
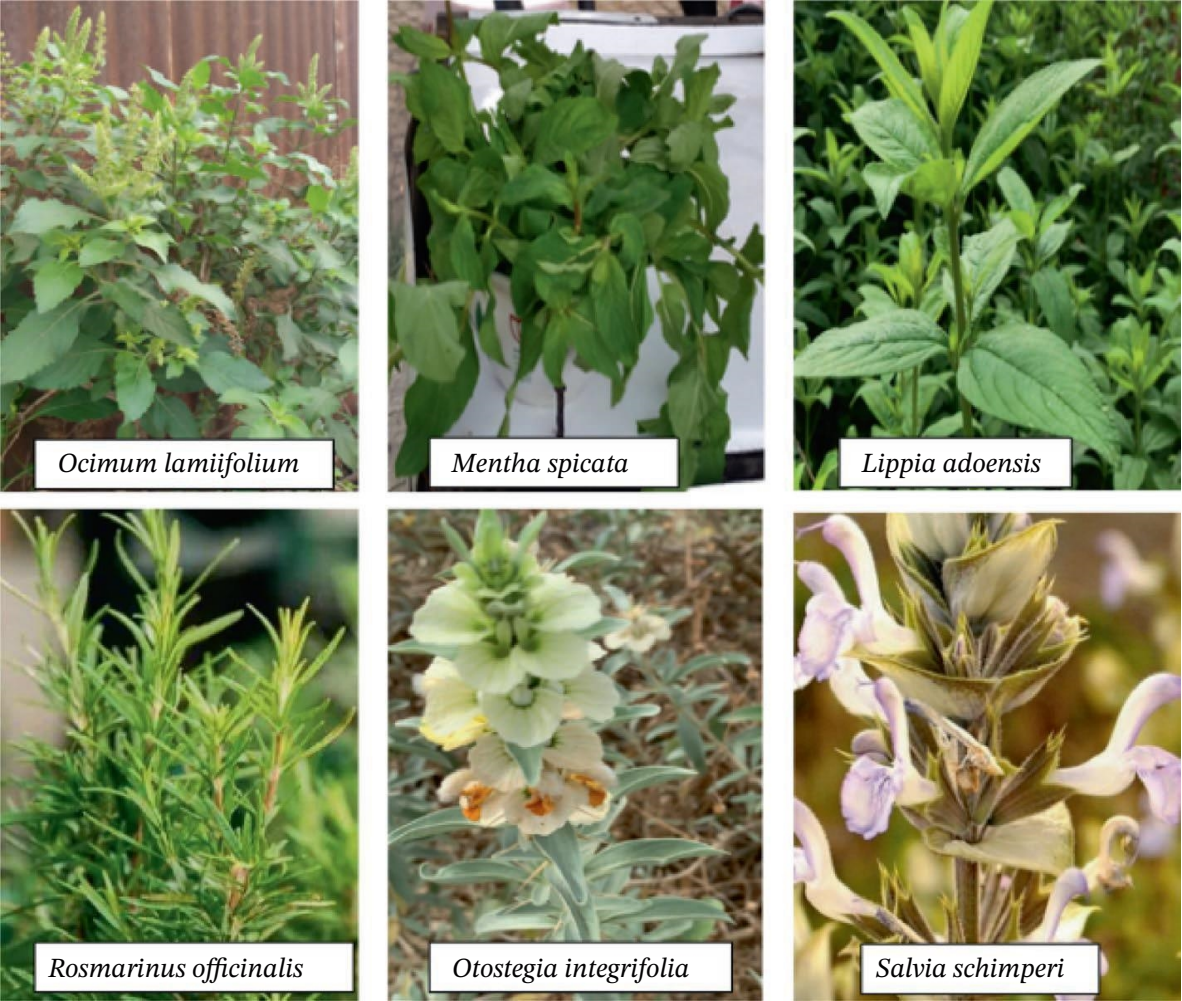
Representative photographs of selected Lamiaceae species collected from Bure Zuria District, Amhara, Ethiopia, during the 2024 growing season.

Medicinal plants were collected from July to September from wild habitat because this season is ideal, as many plants accumulate secondary metabolites in preparation for seed production [32]. The plant components were transported to Bahir Dar University′s extraction laboratory after being packed in a paper bag. All the plant materials were collected after consulting the local traditional healers in the area. Candidate plants were identified using taxonomical keys from the Flora of Ethiopia and Eritrea [33, 34], the useful trees and shrubs of Ethiopia [35], as well as Plants of the World Online (https://powo.science.kew.org). Yitayih Dessie identified all plant species specimens using taxonomic references (Figure 2). Finally, a botanist confirmed the therapeutic plants, which were then kept at the Injibara University Herbarium under their voucher number (Table 1).

**Table 1:** Medicinal plants used for biocontrol investigation.

Botanical name	Local name (Amharic)	Geographic location	Voucher number
*Lippia adoensis* Hochst. ex. Walp.	Koseret	10°18 ^′^2 ^″^N−10°49 ^′^27 ^″^N and 36°52 ^′^2 ^″^E−37°7 ^′^6 ^″^E	YD021
*Mentha spicata* L.	Nana	10°18 ^′^4 ^″^N−10°49 ^′^20 ^″^N and 36°52 ^′^5 ^″^E−37°7 ^′^1 ^″^E	YD005
*Ocimum lamiifolium* Hochst. ex. Benth.	Damakesie	10°18 ^′^8 ^″^N−10°49 ^′^25 ^″^N and 36°52 ^′^6 ^″^E−37°7 ^′^7 ^″^E	YD016
*Rosmarinus officinalis* L.	Siga–Metbesha	10°18 ^′^11 ^″^N−10°49 ^′^17 ^″^N and 36°52 ^′^4 ^″^E−37°7 ^′^0 ^″^E	YD023
*Otostegia integrifolia* Benth.	Tunjut	10°18 ^′^10 ^″^N−10°49 ^′^14 ^″^N and 36°52 ^′^6 ^″^E−37°7 ^′^8 ^″^E	YD012
*Salvia schimperi* Benth.	Yahya Joro	10°18 ^′^6 ^″^N−10°49 ^′^22 ^″^N and 36°52 ^′^7 ^″^E−37°7 ^′^4 ^″^E	YD027

### 2.2. Preparation of Extracts

**Figure 3:**
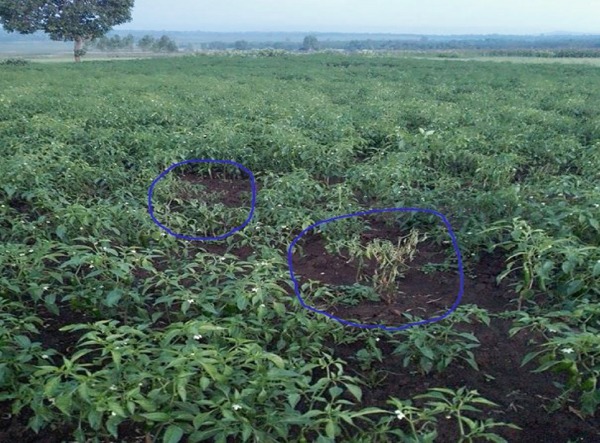
Field symptoms of bacterial wilt caused by *R. solanacearum* in hot pepper cultivation within Ethiopia′s agricultural landscape.

The plant materials were properly washed with tap water and rinsed with distilled water before being air‐dried to constant weight at room temperature under shade and chopped to a suitable size. The dried plant components were pulverized finely with a mechanical grinder. The powder was sieved through 0.6‐mm mesh and stored in polythene bags at 4°C. Since the local traditional healers preferred water as a solvent, aqueous 80% ethanol was used to effectively extract bioactive compounds from medicinal plant materials. These extracts serve as a biocontrol agent for managing severe infections in Ethiopian hot pepper crops (Figure 3). The rationale is that aqueous‐alcoholic (80% ethanol) extracts are better in phytochemicals (e.g., phenolics, alkaloids, flavonoids, and tannins) and antibacterial action [36]. The extraction was performed by macerating 100 g of powdered plant parts in 500 mL of 80% ethanol and continuously shaking for 24 h using an orbital shaker with a magnetic stirrer [37]. The mixture was filtered via Whatman Number 1 filter paper. The extracts were subsequently concentrated at 45°C with a rotary evaporator. The organic solvent extracts were further evaporated to dryness at 40°C in an oven before storing at 4°C for future use.

**Figure 4:**
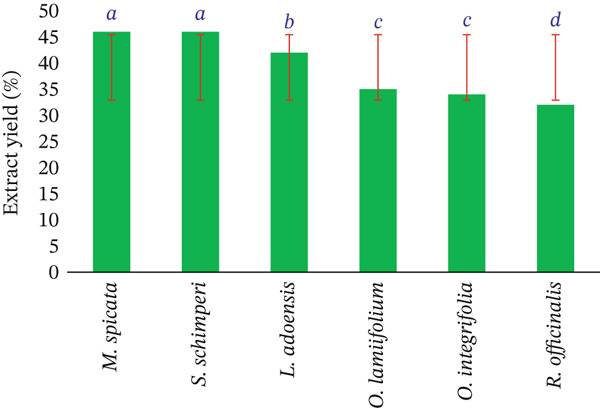
Total percentage yield (*w*/*w*) of crude leaf extracts from Lamiaceae species obtained via ethanol extraction. Yields are calculated based on the initial dry weight of the plant material.

As shown in Figure 4, the extract yield was calculated gravimetrically using the extract′s dry weight, and the initial weight of the leaf powder is as follows:

(1) Extract yield=dry weight of extractinitial powder weight×100%

### 2.3. Phytochemical Screening

#### 2.3.1. Qualitative Phytochemical Screening

Ethanol extracts were used for preliminary screening of phytochemicals such as alkaloids, anthraquinones, glycosides, flavonoids, phenols, saponins, steroids, tannins, and terpenoids according to the standardized methods [38, 39]. The results were expressed as (+) for the presence and (−) for the absence of phytochemical compounds.

#### 2.3.2. Quantitative Phytochemical Analysis

##### 2.3.2.1. Total Flavonoid Content (TFC).

Aluminum chloride complex‐forming assay was used to determine the TFC of the extracts. Catechin was used as a standard to make the calibration curve, and the flavonoid content was determined as catechin equivalents [40]. A standard catechin solution (20, 30, 40, 50, and 60 ppm) was prepared. One milliliter of each extract, 5 mL of distilled water, 0.3 mL of sodium nitrite solution, 0.3 mL of aluminum chloride, 2 mL of 1 mol/L NaOH, and distilled water were added to a 10‐mL volumetric flask and mixed well. The blank solution was prepared in a similar way by replacing aluminum chloride with distilled water. Finally, the absorbance of the blank samples and all extracts was recorded at 510 nm in a UV‐visible spectrophotometer. The TFC of each extract was calculated as catechin equivalents (mg CE/g).

##### 2.3.2.2. Total Phenolic Content (TPC).

The TPC of each extract was determined by using the Folin–Ciocalteu method [41]. A series of standard gallic acid solutions (20, 30, 40, 50 and 60 ppm) were prepared. One milliliter of sample was added into 1 mL of Folin–Ciocalteu phenol reagent and then incubated for 5 min. The produced solution was mixed with 10 mL of 10% Na_2_CO_3_ and 13 mL of deionized water. Then, the solution was shaken and incubated in a dark room, and the temperature was set at 23°C. The absorbance of the solution was measured with a UV‐VIS spectrophotometer at 765 nm. The TPC was then determined by comparing it with a standard curve of gallic acid, and the total phenolic acid was indicated by the value of mg GAE/g of dry sample [42].

### 2.4. Infected Hot Pepper Sample Collection

Infected hot pepper plant samples were collected from September to October 2024 at Bure Zuria District, one of the key growing regions in the Amhara Regional State, as well as Ethiopia. Sampling sites were chosen based on the prevalence of wilting symptoms observed in the hot pepper plants (Figure 3). A site exhibiting both widespread occurrence and significant severity of symptoms was selected for sample collection. Infected plants were carefully uprooted and placed in separate paper bags. To eliminate soil particles and debris, the samples were thoroughly washed with water. The collected samples were then stored in an icebox (4°C) and transported to the Bahir Dar University laboratory room.

#### 2.4.1. Isolation and Identification of *R. solanacearum*

**Figure 5:**
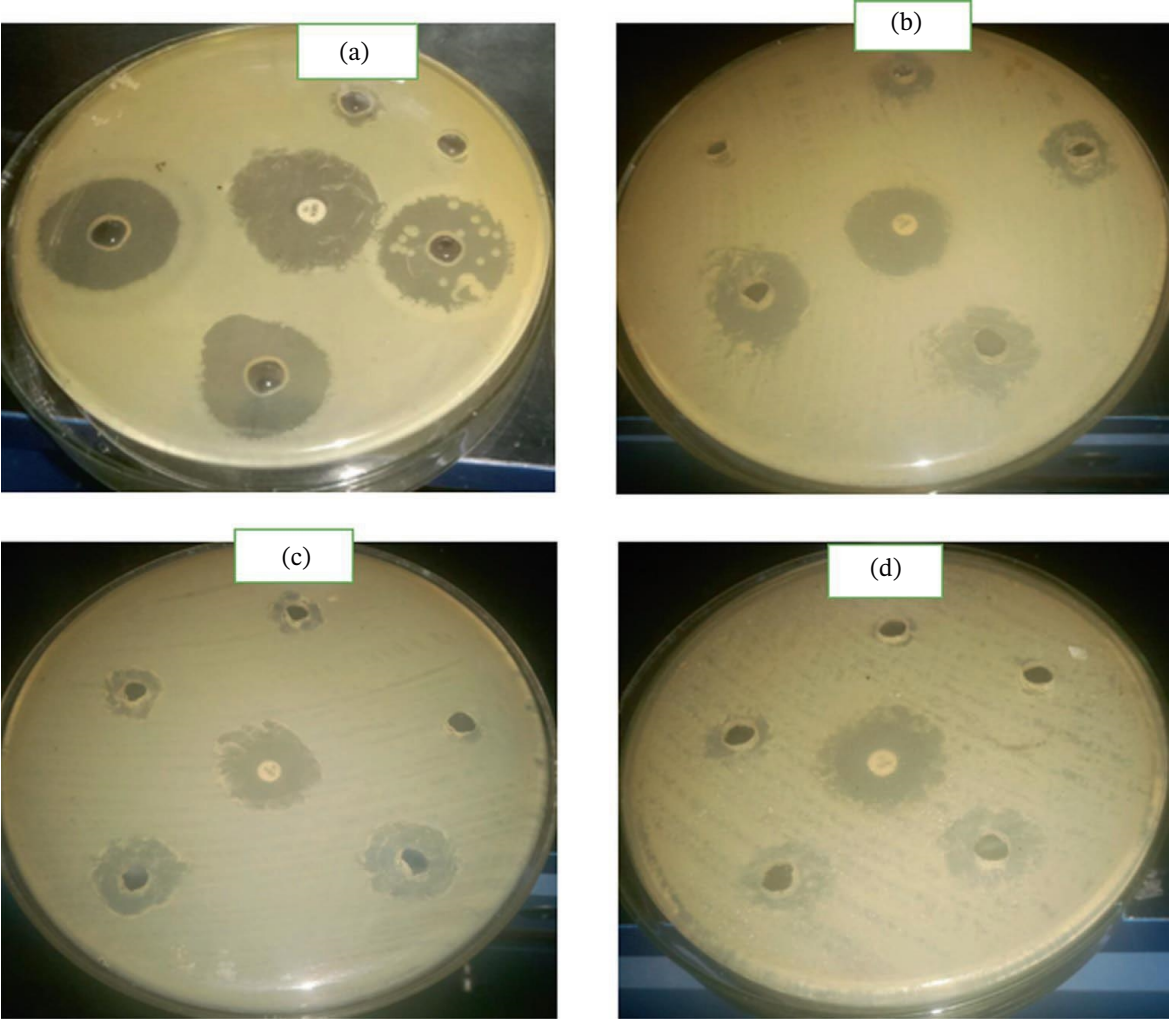
Images of Petri plates showing growth inhibition zones: (a) *M. spicata*, (b) *S. schimperi,* (c) *L. adoensis,* and (d) *O. lamiifolium*.

The collected infected hot pepper plants were surface sterilized by soaking in 5% sodium hypochlorite solution and then rinsed with distilled water [43]. Infected plant parts were cut into small pieces and macerated with a mortar and pestle in the presence of 0.85% NaCl sterile saline solution. Serial dilutions were prepared from the bacterial suspension and inoculated to separate Petri plates poured with casamino acid–peptone–glucose (CPG) growth medium. The Petri plates were incubated at 28^°^C ± 1^°^C for 72 h. After this period, a suspension was prepared from isolated white creamy colonies and stored in screw‐capped vials at 4°C. The bacterial suspension was streaked onto CPG agar medium and incubated to allow for colony development (Figure 5) Successive subculturing was performed until pure, isolated cultures of *R. solanacearum* were established. The isolate was regularly renewed by plating on CPG medium. The pathogen was identified based on colony characteristics and biochemical tests.

Various biochemical tests were conducted to characterize *R. solanacearum*. The Gram staining test was performed, confirming that the isolated bacterium was Gram‐negative. Colonies of *R. solanacearum* were aseptically removed from Petri plates using an inoculating wire loop and placed on a glass slide with a drop of 3% KOH solution. The mixture was stirred for 10 s and observed for the formation of slime threads, following the methodology of recent studies [44, 45].

The catalase test was performed by mixing a loopful of fresh bacterial culture with two drops of 3% H_2_O_2_ solution on a microscope slide. The presence of bubbles indicated a positive catalase test, as described in contemporary literature [44, 45].

For starch hydrolysis, nutrient agar plates containing 0.2% soluble starch (*w*/*v*) were streaked with *R. solanacearum* suspension and incubated at 28^°^ *C* ± 1^°^ *C* until heavy growth was observed. The plates were then flooded with iodine solution (KI), and the formation of a clear zone around a colony indicated a positive reaction for starch hydrolysis [45].

**Figure 6:**
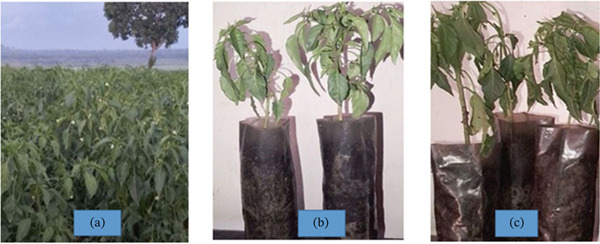
Pathogenicity testing of *R. solanacearum*: (a) hot pepper plants cultivated in the field; (b) hot pepper seedlings grown in the laboratory; and (c) infected hot pepper seedling following bacterial inoculation.

To fulfill Koch′s postulates, a pathogenicity test for *R. solanacearum* was performed as per [46]. Hot pepper seeds were grown in plastic pots for 3 1/2 months. Seedlings were inoculated with 10 mL of a bacterial suspension (1.5 x 10^8^ CFU/mL) around the roots, whereas control plants received sterilized water. Plants were monitored for wilting symptoms over 6 weeks, with any plant showing at least one wilted leaf classified as wilted (Figure 6). The proportion of wilted plants was recorded weekly. After symptom development, the pathogen was reisolated on CPG agar medium, confirming similarity to the original isolates.

### 2.5. Antibacterial Susceptibility Testing of Crude Leaf Extracts

#### 2.5.1. Inoculum Preparation

The bacterial strain (*R. solanacearum*) was isolated from infected hot peppers and maintained on CPG broth slants at 4°C [47]. Pure cultures of the isolates were obtained by subculturing in CPG medium. Inoculum suspension was prepared by picking colonies and diluting them with distilled water. The contents of the test tube were then thoroughly shaken with a vortex until they formed a homogenous suspension. The standard inoculum suspensions were adjusted to turbidity comparable to 0.5 McFarland standards and modified to give a density of 1 × 10^8^ cells or spores/mL [47]. The absorbance of the inoculum was measured at 600 nm using a spectrophotometer (LT‐22313, India) and calibrated to 0.132.

#### 2.5.2. Preparation of Stock Solutions

The extract stock solutions were prepared by dissolving 500 mg in 5 mL of 10% dimethyl sulfoxide (DMSO) [48]. Gentamicin (often used in agricultural practices for the control of bacterial wilt) (30 mg/100 mL sterile distilled water) was used as a positive control [49]. A 10% DMSO solution served as the negative control [50].

#### 2.5.3. Antibacterial Activity Assay

Antimicrobial susceptibility testing was done by the agar well diffusion method according to the Clinical and Laboratory Standards Institute [51, 52]. Approximately 0.5 mL of the bacterial suspension was aseptically inoculated to CPG medium and uniformly distributed using a cotton swab. Reservoir wells were formed by cutting out cylindrical plugs from the solidified CPG medium at equidistant points (30 mm), using a sterile cork borer (diameter: 10 mm) [53]. On each Petri dish, the respective wells were separately filled with the extracts of selected medicinal plants at serial test concentrations of 50, 25, 12.5, and 6.25 mg/mL from a stock concentration of 100 mg/mL, gentamicin 0.3 mg/mL (30 *μ*g/well), and 10% DMSO. The inoculated Petri dishes with test solutions in wells were allowed to diffuse for 30 min before overnight (18 h) incubation at 28^°^C ± 1^°^C. All determinations were done in triplicate. The antimicrobial activity was recorded as the diameter (mm) of the zone of inhibition after incubation [49].

#### 2.5.4. Minimum Inhibitory Concentration and Minimum Bactericidal Concentration

The extracts′ MIC and MBC were measured using the p‐iodonitrotetrazolium (INT) chloride colorimetric test based on broth microdilution method [54]. The test was performed in accordance with the Clinical Laboratory Standards Institute′s recommendations [51]. Bacteria were subcultured on CPG growth media and incubated at 28^°^C ± 1^°^C for 24 h. The bacterial sample was mixed evenly and diluted to satisfy the turbidity criterion of 0.5 McFarland (1 × 10^8^ CFU/mL). The final inoculum concentration (5 × 10^8^ CFU/mL) was achieved after further dilution in each well. A series of concentrations (6.25, 12.5, 25, 50, 80, and 100 mg/mL) were prepared through serial dilution of the working solution in sterile CPG broth medium.

The test was run in a sterile 96‐well plate. Broth medium (100 *μ*L) was added to each well. Extracts (100 *μ*L) and solvent controls (CPG broth and 10% DMSO) were added to each well. The final column served as a blank, containing only broth. Columns 10 and 11 acted as negative controls, containing only medium and bacterial suspension or media, DMSO, and bacteria, respectively. Each well received 50 *μ*L of test bacteria, with the exception of the last row, which acted as a blank. Plant extract concentrations ranged from 6.25 to 100 mg/mL. The plates were sealed and incubated for 24 h at 28^°^C ± 1^°^C. After 24 h of incubation, 40 *μ*L (0.2 mg/mL) of INT chloride was added to each well and incubated at 28^°^C ± 1^°^C for 30 min.

The MIC of the samples was determined after 30 min of incubation. Viable bacteria converted the yellow color to pink. The MIC represents the lowest concentration of an antimicrobial agent that inhibits visible growth of a microorganism after a specified incubation period. To assess the MBC, 50‐*μ*L aliquots from nongrowing wells in the MIC test were added to 150‐*μ*L broth in the well plate and cultured for 48 h at 28^°^C ± 1^°^C. Then, MBC was found to be the lowest concentration of extracts that did not create a color change when INT was added, as previously described.

### 2.6. Determination of Relative Percentage Inhibition

The relative percentage inhibition of the test extract with respect to positive control was calculated by using the following formula [55].

(2) Relative percentage inhibition of the test extract=X−Y∗100/Z−Y

where

X: total area of inhibition of the test extract.

Y: total area of inhibition of the solvent.

Z: total area of inhibition of the standard drug.

The total area of the inhibition was calculated by using area =*A* = *π* *r* ^2^; where, *r* = radius of zone inhibition.

### 2.7. Statistical Analysis

The experimental data were analyzed as mean ± standard deviation (SD) of the inhibition zone. To determine if the means of selected plant extracts have statistically distinct effects on the test organism, a one‐way ANOVA (like Tukey′s HSD) at the *p* = 0.05 significance level was performed using the SPSS software package Version 26. All experiments were performed in triplicate as independent biological replicates to ensure the reliability of the data.

## 3. Results

### 3.1. Extract Yield

The extract yield is a measure of how efficiently the solvent extracts certain components from the source material. The current investigation discovered that the extract yield varied depending on the medicinal plants studied. Figure 4 shows *Mentha spicata* and *Salvia schimperi* leaves yielded the most extract (46%), followed by *Lippia adoensis* (42%), whereas *Rosmarinus officinalis* produced the least (32%).

### 3.2. Qualitative Preliminary Phytochemical Analysis

The ethanol extracts of all selected medicinal plants proved positive for flavonoids, phenolics, and terpenoids. Furthermore, *M. spicata* had all of the examined secondary metabolites, whereas *S. schimperi* was negative for just saponin. However, *R. officinalis* did not contain alkaloids and steroids (Table 2).

**Table 2:** Qualitative phytochemical screening test of plant leaf extracts.

Phytochemical	Test reagent	*M. spicata*	*S. schimperi*	*L. adoensis*	*O. lamiifolium*	*O. integrifolia*	*R. officinalis*
Flavonoid	Alkaline test	++	+	++	++	+	+
Alkaloid	Wagner′s test	+	+	+	++	+	−
Phenols	Ferric chloride test	++	++	++	+	++	++
Tannin	Ferric chloride test	+	+	−	+	+	−
Terpenoid	Salkowski′s test	+	+	++	+	+	+
Saponin	Foaming test	+	−	+	−	+	+
Glycoside	Alkaline test	+	+	+	+	−	+
Anthraquinone	Borntrager′s test	+	+	−	+	+	+
Steroid	Salkowski′s test	+	+	+	+	−	−

*Note:* Key: + = present and − = absent.

### 3.3. Quantitative Determination of Phytochemicals

#### 3.3.1. TPC and TFC

Table 3 shows that *M. spicata* had the highest TPC (184.5 ± 0.5 mg GAE/g dry sample), followed by *S. schimperi* (180.6 ± 0.51 mg GAE/g dry sample), whereas *R. officinalis* had the lowest (122.3 ± 0.23 mg/g dry sample). The ethanol extract of *S. schimperi* has the highest TFC (144.3 ± 0.3 mg CE/g dry weight sample), followed by *M. spicata* (142.4 ± 0.4 mg CE/g dry weight sample), and the lowest was observed in *R. officinalis* (120.5 ± 0.5 mg CE/g).

**Table 3:** Total phenolic (TPC) and total flavonoid (TFC) leaf extracts.

Extracted spp.	Total phenolic contents (mg GAE/g)	Total flavonoid contents (mg CE/g)
*M. spicata*	184.5 ± 0.5	142.4 ± 0.4
*S. schimperi*	180.6 ± 0.51	144.3 ± 0.3
*L. adoensis*	176.7 ± 0.64	138.4 ± 0.45
*O. lamiifolium*	162.3 ± 0.3	134.1 ± 0.1
*O. integrifolia*	134.2 ± 0.26	128.3 ± 0.25
*R. officinalis*	122.3 ± 0.23	120.5 ± 0.5

### 3.4. Antibacterial Susceptibility Testing of Crude Leaf Extracts

The in vitro antibacterial testing of the six (6) medicinal plants showed significant variation among plant species and test concentrations against *R. solanacearum.* Medicinal plant extracts showed a dose‐dependent progressive increase in the growth inhibition zone. The crude extracts showed variation in the mean inhibition zone diameter on *R. solanacearum* (Figure 5). The highest antibacterial activity was observed from *M. spicata* extract (18.33 ± 0.57 mm at 50 mg/mL), followed by *S. schimperi* (16 ± 1 mm at 50 mg/mL), and the lowest growth inhibition zone (3.33 ± 0.57 mm at 6.25 mg/mL) was observed from extracts of *R. officinalis* (Table 4).

**Table 4:** Mean inhibition zone of ethanol extract of medicinal plants against *R. solanacearum.*

Concentration (mg/mL)	Mean inhibition zone (mm)						Gentamicin	10% DMSO
	*M. spicata*	*S. schimperi*	*L. adoensis*	*O. lamiifolium*	*O. integrifolia*	*R. officinalis*		
50	18.33 ± 0.57^a, 1^	15.16 ± 0.76^b, 2^	15.33 ± 1.57^c, 2^	15 ± 0^d, 2^	14.16 ± 0.28^e, 2^	14.16 ± 0.28^f, 2^	24.33 ± 0.28	NI
25	16 ± 1^a, 2^	12.33 ± 0.57^c, 3^	14 ± 0^c,2 3^	13.33 ± 1.15^d, 3^	10 ± 0^f, 4^	11 ± 0^g, 3, 4^		
12.5	11 ± 0^b, 3^	11.33 ± 0.28^c, 3^	9.16 ± 0.28^d, 4^	8.16 ± 0.76^e, 4^	7.66 ± 1.15^f, g, 4^	10 ± 1^g, 3, 4^		
6.25	8 ± 1^c, 4^	6.83 ± 0.28^d, 4, 5^	6 ± 0^e, 4, 5^	5.66 ± 0.57^f, 5^	4.66 ± 0.28^g, 5^	3.33 ± 0.57^h, 5, 6^		

*Note:* Mean values in a column indicated by different letters and mean values in a row indicated by different numbers are significantly different at *p* < 0.05, whereas NI stands for no inhibition zone observed.

Table 4 shows there was a significant difference in the zone of inhibition among tested plant extracts (*p* < 0.05). The standard drug (gentamicin) significantly inhibited the growth of *R. solanacearum.* However, the negative control (10% DMSO) showed no antibacterial activity. Mean values in a column indicated by different letters and mean values in a row indicated by different numbers are significantly different at *p* < 0.05, whereas NI stands for no inhibition zone observed.

#### 3.4.1. Minimum Inhibitory Concentration and Minimum Bactericidal Concentration

**Figure 7:**
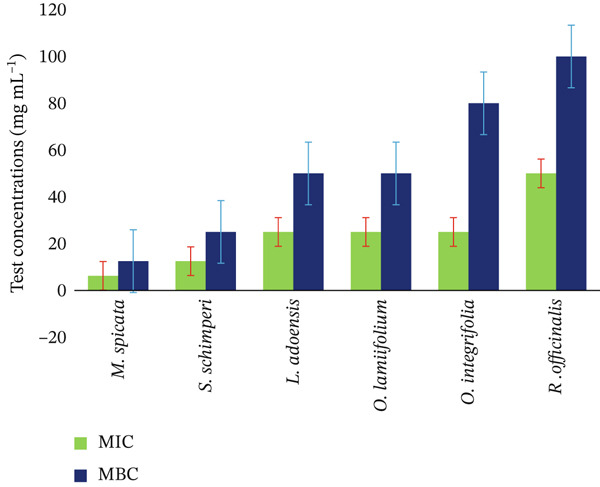
Comparative analysis of the minimum inhibitory concentration and minimum bactericidal concentration of Lamiaceae leaf extracts against *R. solanacearum.*

The efficacy of the extracts was also evaluated using the MIC and MBC. Figure 7 shows their MIC ranges from 6.25 to 50 mg/mL, whereas their MBC ranges from 12.5 to 100 mg/mL. *M. spicata* had a lower MIC (6.25 mg/mL), followed by *S. schimperi* (12.5 mg/mL), and both *M. spicata* and *S. schimperi* had a lower MBC (12.5 mg/mL) against *R. solanacearum.*

### 3.5. Relative Percentage Inhibition

**Figure 8:**
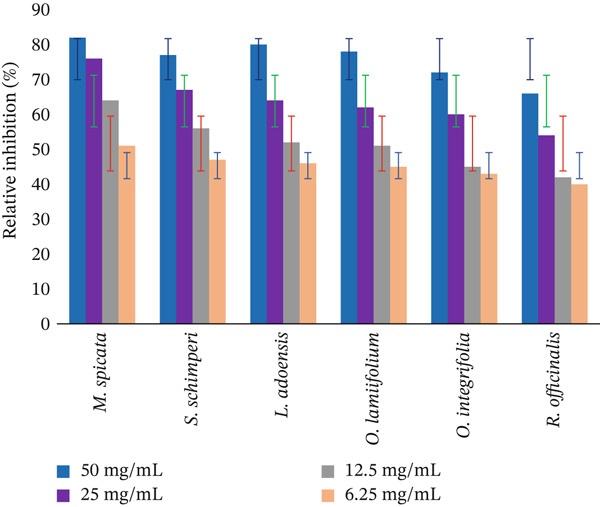
Relative percentage inhibition of Lamiaceae ethanol extracts compared with the positive control (gentamicin, 10 *μ*g/disc) against *R. solanacearum*.

The results of the antibacterial activity of medicinal plant ethanol crude leaf extracts were compared with the positive control, gentamicin (standard drug), for evaluating their relative percentage inhibition (Figure 8). The *M. spicata* ethanol crude extract exhibited maximum relative percentage inhibition at 50 mg/mL (82%), 25 mg/mL (76%), 12.5 mg/mL (64%), and 6.25 mg/mL (51%) test concentrations against *R. solanacearum*. The lowest relative percentage inhibition was obtained from *R. officinalis* at 50 mg/mL (66%), 25 mg/mL (54%), 12.5 mg/mL (42%), and 6.25 mg/mL (40%) test concentration against *R. solanacearum*. The relative percentage inhibition of medicinal plants at each serial test concentration at 25 mg/mL extracts of *M. spicata* compared with *S. schimperi, L. adoensis, O. lamiifolium, O. integrifolia, and R. officinalis* was significantly different at *p* < 0.05.

## 4. Discussion

The use of plant products as novel chemical treatments in crop protection has lately gained traction, and some plant products are now being employed worldwide as eco‐friendly biocontrol agents [56]. The extraction of bioactive components from medicinal plants with 80% ethanol has produced considerable results, particularly for *M. spicata* and *S. schimperi*. Both plants produced significant extraction yields, which can be attributable to a variety of factors, including the extraction method used, solvent concentration, and the plants′ particular phytochemical profiles [57]. Similarly, studies have shown that 80% ethanol solution is very effective for extracting phenolic chemicals and terpenoids from *M. spicata*. This concentration enables the solubilization of both polar molecules, resulting in increased yields of bioactive constituents [58, 59].

The antibacterial and antioxidant properties of medicinal plants are determined by their phytochemical contents. This is related to the fact that medicinal plants′ phytochemical contents are linked to antioxidant and antibacterial properties [60, 61]. All currently examined medicinal plants′ ethanol extracts tested positive for flavonoid, phenolics, and terpenoids. Phenolic chemicals are well‐known for their high antioxidant and antibacterial activity [62]. They have the ability to break bacterial cell membranes and prevent pathogens like *R. solanacearum* from growing [63]. Alkaloids are another class of phytochemicals that can be effectively extracted using ethanol in this study [64]. These compounds have demonstrated significant antibacterial properties and can interfere with bacterial metabolic processes, making them effective against various pathogens, including *R. solanacearum* [65]. Terpenoids, which are also extracted using ethanol, exhibit a broad spectrum of antimicrobial activity. They can disrupt the integrity of bacterial membranes, leading to cell death. Studies have shown that terpenoids can effectively inhibit the growth of *R. solanacearum* [66]. The increasing resistance of *R. solanacearum* to conventional antibiotics necessitates the exploration of alternative antimicrobial agents. Ethanol extracts from various plants, rich in bioactive phytochemicals, may offer a sustainable solution.

The high TPC and TFC observed in the ethanolic extracts of the tested medicinal plants may be attributed to the high polarity of the solvent used [67]. Ethanol is known to be more effective at extracting polar phytochemicals compared with less polar solvents, a finding consistent with previous studies [68–70]. Most flavonoid compounds are polar, and they are mostly extracted by more polar solvents. Previous studies showed that the TFC varied in different solvents depending on their polarity, and the result was an increase in the polarity of the solvent [71–74]. This result is consistent with prior findings on the TPC of plant extracts [62, 75]. Understanding these plants′ phenolic and flavonoid profiles can help guide sustainable agriculture practices, supporting the production of medicinal and antioxidant‐rich species.

The antibacterial activity of selected medicinal herbs against *R. solanacearum* demonstrates their promise as natural alternatives to chemical pesticides, with a dose‐dependent gradual increase in the growth inhibition zone. This implies that the concentration of bioactive compounds increases with dose [76]. *M. spicata* showed the highest inhibitory zone at 50 mg/mL, significantly different from the other five plants examined (*p* < 0.05). This is highly corroborated by our preliminary and quantitative phytochemical results, since *M. spicata* tested positive for all secondary metabolites and had the highest TPC and TFC. The crude ethanol extracts of selected traditional medicinal herbs have substantial antibacterial activity against *R. solanacearum*. Plant secondary metabolites, such as alkaloids, anthraquinones, flavonoids, phenols, saponins, tannins, steroids, and terpenoids, have considerable antibacterial properties [77, 78]; hence, the extracts used in this experiment contained these secondary metabolites. Total phenolics, total flavonoids, total tannins, and total anthocyanins may account for the greatest biological activity [79].

Our findings confirm the well‐documented antibacterial activity of alkaloids and phenolics, as evidenced by the observed inhibition zone sizes. Larger zones indicate more effective antibacterial action, which corresponds with higher concentrations of these phytochemicals in our extracts. For example, alkaloid‐rich extracts produced significantly larger inhibition zones against specific bacterial strains, highlighting a direct relationship between compound concentration and antibacterial efficacy. This correlation reinforces the importance of alkaloids and phenolics in inhibiting bacterial growth and clarifies their contribution to the antimicrobial activity of the extracts.

Some plant extracts showed moderate inhibition despite high flavonoid and phenolic content, highlighting the need to explore potential synergistic effects among phytochemicals. Such synergy might enhance antibacterial activity beyond the contributions of individual compounds. For example, interactions between flavonoids and phenolics could result in a more potent antimicrobial effect, even if their individual impacts seem modest. Future research should focus on these interactions to better understand how combined phytochemicals can improve efficacy, potentially leading to more effective natural antimicrobial agents from plant extracts.

There were no statistically significant differences between *L. adoensis* and *O. lamiifolium* extracts at all test doses. This demonstrates that the two plants contain essentially identical phytochemicals that are responsible for antibacterial activities. However, the crude extract of *S. schimperi* showed a significantly lower inhibition zone (*p* < 0.05) across all evaluated plants. Similarly, crude leaf extracts of *R. officinalis* had significantly lower antibacterial activity than extracts from the other plants at 6.25 mg/mL. Compared with gentamicin, extracts of selected medicinal herbs had significantly lower antibacterial activity (*p* < 0.05). This suggests the need for additional extraction procedures, solvents, and higher test concentrations.

The MIC and MBC are two essential metrics used to assess the antibacterial activity of medicinal plants against bacteria. The MIC represents the lowest concentration of an antimicrobial agent that inhibits visible growth of a microorganism after a specified incubation period, whereas the MBC is the lowest concentration that effectively kills a particular bacterium. It is frequently determined by subculturing samples from the MIC test onto an agar plate to determine viability. *M. spicata* had a lower MIC, whereas *M. spicata* and *S. schimperi* had the lowest MBC. This could indicate that *M. spicata* is extremely effective against the tested bacterial strains. Tura et al. [80] explained that plants had varying degrees of MIC and MBC against the tested pathogen due to differences in plant bioactive constituents and the ability of the tested pathogen to respond to plant extracts. Low MIC and MBC values for other bacteria indicate that the plant extract is effective [81].

In comparing the MIC and MBC values obtained in our study with those reported in previous research, it becomes evident that our findings present a notable advancement in understanding antimicrobial efficacy. Previous studies have documented MIC/MBC values that often reflect a range that may vary significantly depending on the bacterial strain and experimental conditions. For instance, although earlier work indicated MIC values for similar pathogens at concentrations upwards of 75 and 175 mg/mL, respectively [82], our results demonstrated significantly lower values of 6.25 and 12.5 mg/mL, suggesting enhanced potency of the tested medicinal plants. This discrepancy not only underscores the novelty of our findings but also indicates potential improvements in formulation or application methods that could lead to more effective therapeutic options. Furthermore, the consistent reduction in MBC values reinforces the possibility of developing more effective antimicrobial agents, aligning with the ongoing quest for alternatives amidst rising antibiotic resistance.

The antibacterial activity and relative percentage inhibition of ethanol leaf extracts of *M. spicata, S. schimperi, L. adoensis, O. lamiifolium, O. integrifolia,* and *R. officinalis* were assessed in the current study. Relative percentage inhibition of 50% or above is considered an active product and warrants further investigation [1]. In this aspect, ethanol extracts of all examined medicinal plant species showed more than 50% relative percentage inhibition at 50 and 25 mg/mL test concentrations, showing that the extracts were highly powerful at these concentrations. Ethanol leaf extracts of M*. spicata* provided more than 50% relative percentage inhibition across all test dosages. As a result, this herb may have high levels of antibacterial compounds, which are utilized to cure hot pepper diseases.

Overall, using plant extracts with established antibacterial characteristics can help reduce bacterial wilt in crops, decreasing dependency on synthetic chemicals and supporting environmentally friendly agricultural practices. It is critical to develop novel active compounds against new targets. As a result, medicinal plant species (typically with low MIC) may have the potential to become a promising natural antibacterial agent for germ suppression in the pharmaceutical business. However, while using plant extracts for medical purposes, it is always necessary to consider safety and toxicity.

To the best of our knowledge, this study is the first to report on the biocontrol potential of these Lamiaceae leaf extracts against bacterial wilt in Ethiopian hot pepper cultivation, providing critical insights into a time‐sensitive agricultural threat. Although these findings are significant, certain limitations warrant consideration: The reliance on polar solvents may have overlooked more efficient nonpolar extraction methods, and pathogen identification was restricted to morphological and biochemical techniques rather than molecular analysis. Addressing these gaps in future research will be essential for a more comprehensive understanding of the bioactive properties and taxonomic resolution of these promising biocontrol agents.

## 5. Conclusion

The Lamiaceae family has substantial biocontrol potential against *R. solanacearum*, the pathogen that causes wilting in Ethiopian hot pepper crops. The antibacterial capabilities of *M. spicata, L. adoensis, O. lamiifolium, S. schimperi, O. integrifolia,* and *R. officinalis* all demonstrated antibacterial activity against *R. solanacearum*, indicating that they could provide an environmentally friendly and sustainable alternative to treating this devastating disease. Plant extracts with the highest TPC and TFC demonstrated strong antibacterial action. *M. spicata* is the most effective biocontrol agent for *R. solanacearum*, with the lowest MIC and MBC. The plant‐based extracts can be formulated into a product that maintains its efficacy over extended storage periods, ensuring their practical use in agriculture. Incorporating these extracts into the soil can bolster beneficial microbial populations that suppress pathogens. Foliar treatments with concentrated extracts can enhance hot pepper defenses, whereas companion planting with pest‐repelling species offers additional protection. These biocontrol strategies have the potential to improve crop resilience, reduce reliance on chemical pesticides, and promote regional agricultural sustainability and food security. Further research is essential to refine these biocontrol techniques, including molecular identification, and to evaluate their effectiveness across various agricultural settings in vivo.

**Nomenclature**
ANOVA: analysis of variance
CFU: colony forming unit
CLSI: Clinical and Laboratory Standards Institute
CPG: casamino acid–peptone–glucose
DMSO: dimethyl sulfoxide
DZI: diameter of zones of inhibition
INT: iodonitrotetrazolium chloride
MBC: minimum bactericidal concentration
MIC: minimum inhibitory concentration
TFC: total flavonoid content
TPC: total phenolic content

## Author Contributions

Y.D. conceived the study, conducted experiments, evaluated data, and wrote both the draft and final manuscript. D.M. and A.T. serve complementary functions in hot pepper species selection and data validation. B.A., B.K., and M.L. monitor the antimicrobial assay, while A.A. helps with data validation and revises the manuscript.

## Funding

No funding was received for this manuscript.

## Disclosure

The final manuscript was read and approved by all authors.

## Ethics Statement

The authors have nothing to report.

## Conflicts of Interest

The authors declare no conflicts of interest.

## Data Availability

The article contains all of the necessary data.
